# Oxidative Stress Response of Honey Bee Colonies (*Apis mellifera* L.) during Long-Term Exposure at a Frequency of 900 MHz under Field Conditions

**DOI:** 10.3390/insects15050372

**Published:** 2024-05-20

**Authors:** Marinko Vilić, Ivona Žura Žaja, Mirta Tkalec, Perica Tucak, Krešimir Malarić, Nato Popara, Nikolino Žura, Selim Pašić, Ivana Tlak Gajger

**Affiliations:** 1 Department of Physiology and Radiobiology, Faculty of Veterinary Medicine, University of Zagreb, 10000 Zagreb, Croatia; mvilic@vef.unizg.hr; 2 Department of Biology, Faculty of Science, University of Zagreb, 10000 Zagreb, Croatia; mirta.tkalec@biol.pmf.hr; 3 State Inspectorate of Republic of Croatia, 10000 Zagreb, Croatia; perica.tucak@gmail.com; 4 Department of Communication and Space Technologies, Faculty of Electrical Engineering and Computing, University of Zagreb, 10000 Zagreb, Croatia; kresimir.malaric@fer.hr; 5 Department of Physics, Faculty of Veterinary Medicine, University of Zagreb, 10000 Zagreb, Croatia; npopara@vef.unizg.hr (N.P.); spasic@vef.unizg.hr (S.P.); 6 Faculty of Veterinary Medicine, University of Zagreb, 10000 Zagreb, Croatia; nikolino.zura@zvu.hr; 7 Department for Biology and Pathology of Fish and Bees, Faculty of Veterinary Medicine, University of Zagreb, 10000 Zagreb, Croatia; itlak@vef.unizg.hr

**Keywords:** radiofrequency radiation, honey bee, antioxidants, field conditions

## Abstract

**Simple Summary:**

Most research on radiofrequency electromagnetic fields (RF-EMFs) in honey bees has studied adult bees under controlled laboratory conditions after direct exposure to devices that generate RF-EMF. To date, there are many uncertainties about the effects of RF-EMFs on different developmental stages of honey bees under field conditions. We investigated oxidative stress in larvae, pupae and adult honey bees after exposure to RF-EMFs originating from phone base station towers under field conditions. The study was conducted on a total of fifteen honey bee colonies exposed to RF-EMFs at a frequency of 900 MHz, divided into three locations with different electric field levels. All the honey bee colonies at the three locations were exposed for one year. Antioxidant parameters such as glutathione-S-transferase, catalase and superoxide dismutase, as well as the formation of thiobarbituric acid reactive substances, were measured in samples of larvae, pupae and the midguts of adult honey bees. Our results show that the activity of antioxidant enzymes changes and that the level of the analyzed parameters depends on the developmental stage of the honey bee, the level of the electric field and the exposure time.

**Abstract:**

In this study, oxidative stress and lipid peroxidation in honey bee larvae, pupae and the midguts of adult bees were investigated during a one-year exposure to radiofrequency electromagnetic fields (RF-EMFs) at a frequency of 900 MHz under field conditions. The experiment was carried out on honey bee colonies at three locations with electric field levels of 30 mV m^−1^, 70 mV m^−1^ and 1000 mV m^−1^. Antioxidant enzymes, glutathione-S-transferase (GST), catalase (CAT) and superoxide dismutase (SOD) and thiobarbituric acid reactive substances (TBARS) as indicators of lipid peroxidation were measured spectrophotometrically. The GST activity within the same developmental stage showed no significant differences regardless of electric field level or sampling time. The highest GST activity was found in the pupae, followed by activity in the larvae and midguts. Both CAT activity and TBARS concentration were the highest in the midguts, regardless of field level and sampling time. The larvae showed a significantly higher TBARS concentration at the location with an electric field level of 1000 mV m^−1^ compared to the locations with lower levels. Our results show that RF-EMFs at a frequency of 900 MHz can cause oxidative stress in honey bees, with the larval stage being more sensitive than the pupal stage, but there was no linear relationship between electric field level and effect in any of the developmental stages.

## 1. Introduction

The modern way of life is undoubtedly conditioned by the use of various achievements of scientific and technological development, among which the application of radiofrequency communication (RFC) is indispensable. Most sources of RFC were already known and used in the 20th century; however, their real expansion occurred in the early 1990s, with the use of mobile phones and wireless communication technologies. In 2022, the number of mobile–cellular subscriptions exceeded the total world population, and there were a reported 8.6 billion mobile phone subscriptions worldwide, with the penetration rate of mobile–broadband subscriptions growing by an enormous 14.8% per year over the last 10 years [1], and the trend continues to grow. The intensity level of radiofrequency electromagnetic radiation fields (RF-EMFs) around a frequency of 1 GHz has increased by about 10^18^ times compared to natural levels [2].

In addition to the wide use of mobile phones and other sources that generate RF-EMFs, public concern about harmful effects on human and animal health is also growing. Indeed, the results of numerous studies have shown that exposure to RF-EMFs at the operating frequency of wireless devices (mobile phones, routers, base stations) may cause various non-thermal biological effects such as oxidative stress [3,4], immune system dysfunction [5,6], genotoxic effects [7,8,9], effects on reproduction [10] and adverse effects on male fertility [11,12]. The mentioned biological effects have been proven in vitro and in vivo on different species of animals including mammals and insects, especially honey bees [13,14,15]. 

The honey bee is one of the most important insects for maintaining balance in natural ecosystems. It is assumed that the honey bee (*Apis mellifera*) plays the most important role in the pollination of all insect species from the order Hymenoptera, as it is involved in almost 80–85% of the pollination of the world’s crops [16,17,18]. In addition to pollination, honey bees are also important for the production of apian products (honey, propolis, pollen, wax, royal jelly and bee venom), which are widely used in human nutrition, medicine and the pharmaceutical industry.

Previous studies on the effects of RF-EMFs on honey bee colonies mostly examined adult honey bees in the laboratory or under unnatural conditions, i.e., after direct exposure to devices emitting radiofrequency electromagnetic radiation such as mobile phones or Wi-Fi networks [19,20,21,22]. Most results showed reduced colony strength and queen laying rate [22,23] as well as the initiation of “worker piping” [19,24,25] associated with swarming. The results of some experiments suggest that RF-EMFs generated by mobile phone base stations or devices equivalent to commercial Wi-Fi devices could actually alter honey bees’ navigational abilities, i.e., reduce foraging success and prevent them from returning to their hives [26,27]. Behavioral changes and biological activities, as well as oxidative stress and short-term memory under laboratory conditions in honey bees, have been reported previously [20,21,28,29].

To date, there remain many uncertainties about the effects of radiofrequency radiation on honey bee colonies, as there is a lack of studies under field conditions. In view of this, Vanbergen et al. [30] recommended more field-realistic studies on the exposure of pollinators to RF-EMFs, and Panagopoulos et al. [31] emphasized that exposure to RF-EMFs in field conditions is an important aspect of the study of biological effects.

How frequency, electric field strength, modulation and exposure duration, as well as the developmental stage, can significantly influence biological responses after RF-EMF exposure [13,32,33,34], and because currently there is a lack of research on the impact of RF-EMFs on oxidative stress in different developmental stages of honey bees after being exposed to RF-EMFs under field conditions, we wanted to investigate the antioxidant parameters (indicators of antioxidant defenses) in honey bee larvae, pupae and adult bees exposed to RF-EMFs under field conditions. In this study, honey bee colonies were exposed to three electric field levels at a frequency of 900 MHz originating from mobile phone base stations: (i) electric field level of 1000 mV m^−1^, the highest value found in the environment; (ii) 30 mV m^−1^, the lowest value of electric field found in the environment and (iii) 70 mV m^−1^, the value measured at the location of the stationary apiary, to which the honey bees were continuously exposed for a long time. We would also like to emphasize that all the electric field levels used in this study correspond to the values found in the natural environment but with a lower probability of the colonies being exposed to the chosen highest measured field level, which is almost 30 times higher than the lowest level.

Since changes in the activity of antioxidant enzymes such as catalase (CAT), glutathione S-transferase (GST) and superoxide dismutase (SOD), as well as the formation of thiobarbituric acid reactive substances (TBARS), as an indicator of oxidative damage were documented in our previous study [29] on honey bees after exposure to RF-EMR under laboratory conditions, the aim of this study was to answer the following questions: (a) Could RF-EMF cause lipid peroxidation and changes in three vital antioxidant enzymes (CAT, GST and SOD) at different developmental stages of honey bee workers under field conditions? (b) Is there a possibility of a long-term effect on oxidative stress in honey bees after one year of exposure?

## 2. Materials and Methods

### 2.1. Honey Bee Colonies and Exposure Conditions

The study was conducted on Carniolan honey bees (*Apis mellifera carnica*, Pollmann, 1879) exposed to RF-EMFs from mobile phone base stations in their natural environment. A total of fifteen (15) honey bee colonies (Langstroth–Root-type hives) were randomly selected and equalized after a clinical inspection.

**Figure 1:**
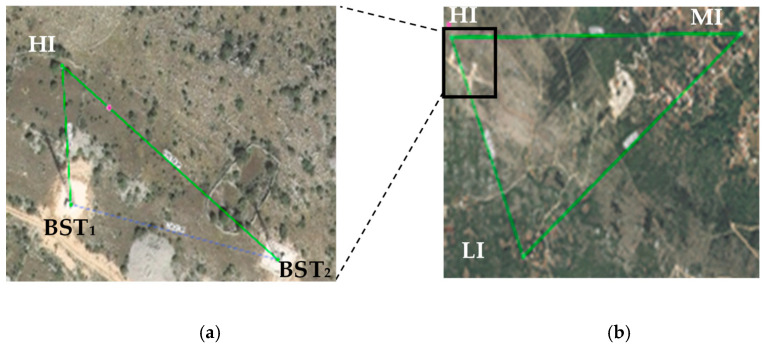
Honey bee colonies (*Apis mellifera*) were exposed to RF-EMFs from mobile phone base stations (BST_1_ and BST_2_) in their natural environment: (**a**) overview of the location of honey bee colonies at the site of the high electric field (HI) and the base station towers (BST_1_ and BST_2_), (**b**) overview of the locations between the honey bee colonies situated at the three sites with different electric field levels: the high electric field (HI), low electric field (LI) and medium electric field (MI).

All the experimental colonies had one-year-old queens of the same genetic origin produced by a registered queen breeder. The experiment was carried out in three different locations: (I) Five (5) honey bee colonies were located near mobile phone base station towers with a frequency of 900 MHz and an average environmental electric field level of 1000 mV m^−1^, named high intensity (HI). The site determinants were: 43°23′7.20″ N; 17°13′16.97″ E; 534 m above sea level; and 67.24 m and 159.76 m distance from the mobile phone base stations (Figure 1a). (II) Five (5) honey bee colonies located at a site with a frequency of 900 MHz and an average environmental electric field level of 30 mV m^−1^, named low intensity (LI), with the site determinants being 43°22′43.55″ N; 17°13′34.55″ E; 450 m above sea level; and 790 and 700 m distance from mobile phone base stations. (III) Five (5) honey bee colonies located at a site with a frequency of 900 MHz and an average environmental electric field level of 70 mV m^−1^, named stationary location or medium intensity (MI). The site determinants were 43°23′717″ N; 17°14′30.05″ E; 277 m above sea level; and 1630 m and 1520 m distance from the mobile phone base stations. The honey bee colonies located at MI were used as a control as these 5 colonies were already stationed at that site before the experiment was carried out, and the other 10 experimental colonies were formed from colonies located at this site.

The distance between the three locations (honey bee colonies), i.e., HI and LI, HI and MI and MI and LI was 829.77 m, 1.64 km and 1.44 km, respectively (Figure 1b). All the honey bee colonies at the three mentioned locations were exposed to the radiofrequency electromagnetic field continuously for one year. The average electric field level and frequency were measured at each site using the portable spectrum analyzer, NARDA SRM 3000 (Narda Safety Test Solutions GmbH, Pfullingen, Germany). All the collected samples originated from the worker brood and worker adults. Samples were taken on three occasions (at 2 weeks, 5 months and 1 year) after the beginning of observation (Table 1). During the experiment, all the honey bee colonies were treated with the same authorized and registered veterinary medicine against varroosis at the same time. 

### 2.2. Sample Preparation and Assays of Oxidative Stress Parameters

Five- to six-day-old larvae, pupae in the stage of purple eyes and the midguts of adult honey bees were collected from each honey bee colony and placed in five Eppendorf tubes (6 larvae/tube; 4 pupae/tube; and 10 midguts/tube). The adult bees were forager bees older than 21 days and were collected by shaking the periphery hive frames. Before the midgut of an adult bee was dissected, the adult bee was briefly exposed to ice. Dissection: we pulled out the posterior segment of the abdomen with fine forceps to remove the intestines from the abdomen. Then, we cut the midgut with a scalpel on a clean surface and placed it in an Eppendorf tube. The Eppendorf tubes containing the samples were frozen in liquid nitrogen (−196 °C) and delivered to the laboratory where they were stored at −80 °C until biochemical analysis. Immediately prior to biochemical analysis, the collected tissues were homogenized in a cold potassium phosphate buffer (50 mM, pH 7.0) containing 0.5 mM EDTA, using QIAGEN’s TissueLyser II apparatus (60 s at 15 Hz) for sample disruption; the resulting homogenate was centrifuged two times (each at 15,000× *g* for 15 min, at 4 °C). The supernatants collected in this process were then used for further biochemical investigations. 

The glutathione S-transferase (GST), catalase (CAT) and superoxide dismutase (SOD) activity, as well as the level of lipid peroxidation (TBARS), were determined in accordance with our previously published study [29,35]. 

In brief, the GST activity (EC 1.8.1.7) was measured via the modified method of [36] using 1-chloro-2,4-dinitrobenzene (CDNB) at 340 nm (ε = 9.6 mM^−1^ cm^−1^). The reaction mixture consisted of 50 μL of the sample, 100 mM of potassium phosphate buffer at a pH of 6.5, 2 mM of CDNB and 2.5 mM of GSH. The results were expressed in units per mg of protein, where one unit was defined as the amount of enzyme that produces 1 µmol of GS-DNB of conjugate per minute under the assay conditions. CAT (EC 1.11.1.6), was measured using the absorbance decrease at 240 nm (ε = 39.4 M^−1^ cm^−1^) as per Aebi [37]. The reaction mixture consisted of 25 μL of the sample and 50 mM of potassium phosphate buffer, at a pH of 7.0, with the addition of 10 mM H_2_O_2_. The CAT activity was presented in units per mg of protein. One unit was defined as the amount of enzyme that hydrolyzes 1 μmol of H_2_O_2_ per minute at 25 °C and a pH of 7.0. For the determination of SOD activity (EC 1.15.1.1), the reaction mixture consisted of 0.5 mM of xanthine and 0.01 mM of cytochrome c in 50 mM of potassium phosphate buffer, at a pH of 7.8, containing 0.01 mM of EDTA. This was used in a modified xanthine oxidase/cytochrome c method, according to McCord and Fridovich [38], after enough xanthine oxidase was added to cause a change in absorbance of 0.025 per min. The results were expressed in arbitrary units, where one unit was defined as inhibiting the rate of cytochrome reduction in the coupled xanthine–xanthine oxidase system by 50% at a pH of 7.8 and 25 °C. The level of lipid peroxidation was measured as the formation of thiobarbituric acid reactive substances (TBARS), a byproduct of lipid peroxidation that reacts with thiobarbituric acid [39]. The supernatants (300 µL) were mixed with 200 µL of cold 20% (*w*/*v*) trichloroacetic acid to precipitate the proteins. The precipitate was pelleted by centrifugation (10,000× *g* for 15 min at 4 °C), and the supernatant obtained was reacted with 400 μL of 1% (*w*/*v*) thiobarbituric acid prepared in 20% TCA. After heating at 95 °C for 30 min, the mixture was cooled in an ice bath. Supernatant absorbance at 532 nm was measured, and the results were corrected for nonspecific turbidity by subtracting the absorbance at 600 nm. The TBARS content, expressed per mg of protein, was obtained using an extinction coefficient of 155 mM^−1^ cm^−1^.

The concentration of protein in the supernatants was determined according to Bradford [40], taking bovine serum albumin as a standard.

### 2.3. Statistical Analysis

All the results were expressed as the mean (M) and standard error (SEM) of 30 larvae, 20 pupae and 50 midguts. A 12 Software package (StatSoft, Inc., Tulsa, OK, USA) was used for the statistical analysis. Normality was tested with the Kolmogorov–Smirnov test. After the Kolmogorov–Smirnov test, the results were tested using an analysis of variance (ANOVA) to determine differences between groups. Multiple comparisons between means were determined using the Tukey HSD test. A statistical difference at *p* < 0.05 was considered significant.

## 3. Results

### Oxidative Stress Parameters

The activity of antioxidant parameters as well as TBARS concentration in the larvae, pupae and midguts of honey bees exposed to RF-EMFs at a frequency of 900 MHz and electric field levels of 70 mV m^−1^, 30 mV m^−1^ and 1000 mV m^−1^ for 2 weeks, 5 months and 1 year are presented in Table 2, Table 3, Table 4 and Table 5. 

The GST activities in all the honey bee samples during exposure to RF-EMFs at a frequency of 900 MHz at locations with different electric field levels (30, 70 and 1000 mV m^−1^) were not statistically different when comparing the results between the different locations at the same observation periods (2 weeks, 5 months or 1 year) or when comparing the results within the same locations (electric field level) at the different observation periods (between 2 weeks, 5 months or 1 year).

The CAT activity in the larvae was statistically increased (*p* < 0.05) at the HI location (1000 mV m^−1^) compared to the MI and LI locations with lower electric fields, i.e., 70 mV m^−1^ and 30 mV m^−1^, at the fifth month of exposure, while it was increased at the LI (30 mV m^−1^) compared to the MI (70 mV m^−1^) and HI (1000 mV m^−1^) locations at 1 year of exposure. The CAT activity in honey bee larvae at the LI location (30 mV m^−1^) was statistically increased (*p* < 0.05) at 1 year of honey bee colony exposure compared to 5 months of exposure. The CAT activity in the pupae was not statistically different when comparing different locations at the same times of observation. The highest CAT activity in the pupae was measured over 5 months of exposure at locations LI (30 mV m^−1^) and MI (70 mV m^−1^), and the values at both locations were significantly higher (*p* < 0.05) when compared with those obtained at 2 weeks of exposure, while at the MI location, it was also higher compared to that at 1 year of exposure. The CAT activity in the midguts of the adult honey bee colony was significantly higher (*p* < 0.05) at the HI (1000 mV m^−1^) compared to the MI (70 mV m^−1^) location after the 2-week exposure as well as the 1-year exposure. On the other hand, it decreased at the HI location (1000 mV m^−1^) compared to the LI (30 mV m^−1^) at the 5-month exposure. The CAT activity in the guts of the bee colony at the location with the highest electric field (1000 mV m^−1^) was the lowest at the 5-month exposure compared to the 2-week and 1-year exposures.

The SOD activities in all the honey bee samples (larvae, pupae and midgut) during exposure to RF-EMF at a frequency of 900 MHz and from locations with different electric field levels (30, 70 and 1000 mV m^−1^) did not significantly differ when the results were compared between the different locations over the same observation period. The exception was the SOD activity in the midguts of the honey bee adults at one year of exposure, which significantly increased (*p* < 0.05) at the HI location, with the highest electric field level (1000 mV m^−1^), compared to the MI location (70 mV m^−1^). Regarding different times of observation, the activity of SOD in the midguts of honey bees at the HI location (1000 mV m^−1^) was significantly lower (*p* < 0.05) at 2 weeks of exposure than at one year of exposure.

In the larvae, the TBARS concentration was significantly enhanced at the HI location (1000 mV m^−1^) compared to the other two locations, MI (70 mV m^−1^) and LI (30 mV m^−1^) after the 2-week exposure, while after the 1-year exposure, the TBARS were also higher at the HI as well as at the LI locations compared to the value obtained at the MI (70 mV m^−1^). The TBARS in larvae of honey bees from the LI location was significantly higher after 1 year of exposure than after 2 weeks of exposure. In pupae, there were no statistical differences between the results when comparing the different locations and the different exposure times. The TBARS concentration in the midguts was statistically increased at the HI location (1000 mV m^−1^) compared to the MI (70 mV m^−1^) after 2 weeks of exposure.

## 4. Discussion

To date, there are very few published studies on the effects of RF-EMFs on insects under field conditions and long-term exposure. The majority of studies were conducted under laboratory conditions and over short-term exposure. Lazaro et al. [41] showed for the first time that different wild pollinator species differ in their abundance during exposure to RF-EMFs from telecommunication antennas in natural habitats on two different Mediterranean islands (Lesvos and Limnos) in the Northeastern Aegean (Greece), with a range of electric field levels from 10 mV m^−1^ to a maximum value of 670 mV m^−1^. The same authors concluded that the result obtained could be due to different sensitivities to RF-EMFs, especially in the larval stage. Currently, there is also a lack of data on the induction of oxidative stress in honey bees during exposure to RF-EMFs. 

In this study, honey bee colonies were exposed to an electric field level of 30 mV m^−1^ as the lowest value of electric field found in the environment, then 70 mV m^−1^ and 1000 mV m^−1^, the highest value found in the environment

We have previously reported that RF-EMFs at 900 MHz affect the antioxidant system of honey bee larvae after short-term exposure under laboratory conditions [29,35]. These previous results showed that the effects strongly depend on the physical properties of the radiofrequency electromagnetic radiation, the measured antioxidant parameters and the experimental setup, as well as there being no linear field-response relationship. The results obtained in this study also confirm the similarity with the previous study. This means that the results obtained in this study, i.e., the activity of the antioxidant enzymes and the concentration of lipid peroxidation products, depend on the developmental stage of the honey bees, the environmental electric field levels and the duration of exposure. Indeed, almost all the significant changes in the observed parameters occurred at the highest field level in the larval stage or adults (midguts of adult workers). 

The results of numerous studies on the effects of RF-EMFs after short-term exposure indicate that electromagnetic radiation at the frequency of mobile telephony can cause the increased formation of reactive compounds, even at low intensity, thus inducing oxidative stress [42,43,44] as well as suppression of the immune system and antioxidant defense mechanisms [45].

The overproduction of reactive oxygen species (ROS) following exposure to RF-EMFs is scavenged by SOD, CAT and GST, the most important ROS-scavenging enzymes in honey bees [46,47,48,49]. One of the reasons for the observed antioxidant enzyme activity at certain developmental stages could be the physiological developmental profile of the antioxidant enzymes studied and their physiological function in honey bees. Namely, it is known that under physiological conditions, the activities of SOD, CAT and GST in a larva increase slightly from the first to the sixth day, and then decrease until the end of honey bee development, with CAT activity decreasing the most [50]. According to the same research, young worker bees have lower antioxidant enzyme activity than pupae, and CAT is the main antioxidant enzyme responsible for the degradation of hydrogen peroxide in the honey bee brood. Although, in this study, the results of enzyme activity were not compared between the different developmental stages, different levels of the enzymes tested were also found, i.e., the highest GST activity was found in the pupae, followed by the larvae and midguts, regardless of field level and time of sampling. In addition, both the CAT activity and TBARS concentration were highest in the midguts, and GST activity was lowest, regardless of field level and time of sampling, and they followed in the larvae and pupae. In addition, Weirich et al. [46] studied the activity of antioxidant enzymes (CAT, SOD and GST) in the hemolymph, midgut, pectoral muscle and sperm in spermatheca and found that the activity of the antioxidants studied was differently distributed in the tissues of worker, unfertilized and fertilized queen bees. Indeed, the same authors found that the activity of CAT and GST was significantly higher in the midgut than in the muscle and hemolymph in all members (castes) of the honey bee colony, while the activity of SOD did not show significant differences between the tissues and bee groups studied. 

Based on our results, where the levels of TBARS (at two weeks and one-year exposure) and CAT (at 5 months and one-year exposures) were significantly increased in the larvae, we hypothesize that the larvae are more sensitive to RF-EMF exposure than the pupae. The higher TBARS content could be explained by the lipid content of the larvae, which have a significantly higher fat content than the pupae [51] and therefore react more sensitively to oxidative stress. In contrast, in the developmental stage of the pupae, no statistical difference could be detected for any of the indicators examined between different locations with different electric field levels at all three sampling times. One possible reason for this is the fact that the pupa stage is able to maintain an oxidation–reduction equilibrium at this stage of frame development due to the higher physiological activity of defense enzymes, thus overcoming the possible cell damage caused by oxidative stress. The CAT, SOD and GST activity showed no linearity in relation to the field level and the time of sampling. However, if we compare the parameters obtained only in the midguts of the adult bees, we find that the greatest changes were recorded at the location with the highest electric field level (1000 mV m^−1^). These findings indicate that the honey bees located at a higher field level are exposed to higher oxidative stress than the honey bee colonies located at a lower electric field level, which can also explain the results of our previous work [28], in which the authors demonstrated increased aggressiveness and restlessness of adult bees and an accelerated process of silent queen replacement in the colonies located in a field level of 1000 mV m^−1^. 

## 5. Conclusions

In conclusion, our results indicated that exposure to RF-EMFs at a frequency of 900 MHz in field conditions may cause oxidative stress in certain developmental stages of honey bees. Most of the significant changes occurred in the second week of the exposure at the location with the highest electric field level, and there was no linear relationship between field level and effect in any of the developmental stages. Among antioxidative enzymes, CAT showed the greatest change and SOD the least change in activity. In addition, the antioxidant profile could serve as a bioindicator of oxidative stress in bees during exposure to RF-EMFs, especially CAT activity and TBARS concentration in the larvae and midguts of the adult honey bees. Since RF-EMF mechanisms are not yet well known, it is difficult to say what consequences such radiation could have on their physiological characteristics. Therefore, our results show the need for further research in the developmental stages of honey bees, including not only oxidative stress parameters but, as far as possible, expression of the gene for the antioxidant enzymes, genotoxic parameters and a greater number of variations of RF-EMF sources in the natural conditions.

## Figures and Tables

**Table 1:** Meteorological conditions and external temperatures during sampling of worker bee larvae, pupae and midguts of adult workers.

Sampling Time	Weather Conditions	Temperature on Sampling Day
2 weeks (April)	Cloudy	18 °C
5 months (September)	Clear	24 °C
1 year (April)	Clear	17 °C

**Table 2:** Glutathione S-transferase (GST) activity in the larvae, pupae and midguts of honey bees exposed to RF-EMFs at a frequency of 900 MHz and electric field levels of 70 mV m^−1^ (medium intensity—MI), 30 mV m^−1^ (low intensity—LI) and 1000 mV m^−1^ (high intensity—HI) for 2 weeks, 5 months and 1 year.

Sampling Time	Location	Electric Field Level (mV m^−1^)	GST (Unit mg^−1^ _proteins_)		
			Larvae (N = 30)	Pupae (N = 20)	Midguts (N = 50)
2 weeks	MI	70	0.16 ± 0.02 ^ab^	0.18 ± 0.004 ^b^	0.04 ± 0.003 ^ab^
	LI	30	0.18 ± 0.004 ^ab^	0.26 ± 0.03 ^ab^	0.04 ± 0.01 ^ab^
	HI	1000	0.17 ± 0.02 ^ab^	0.25 ± 0.01 ^ab^	0.05 ± 0.004 ^a^
5 months	MI	70	0.15 ± 0.01 ^a^	0.24 ± 0.02 ^ab^	0.04 ± 0.003 ^ab^
	LI	30	0.16 ± 0.01 ^ab^	0.29 ± 0.02 ^a^	0.04 ± 0.002 ^b^
	HI	1000	0.19 ± 0.01 ^ab^	0.27 ± 0.03 ^a^	0.04 ± 0.001 ^ab^
1 year	MI	70	0.18 ± 0.02 ^ab^	0.21 ± 0.01 ^ab^	0.03 ± 0.003 ^b^
	LI	30	0.18 ± 0.01 ^ab^	0.26 ± 0.01 ^ab^	0.04 ± 0.002 ^b^
	HI	1000	0.21 ± 0.02 ^b^	0.23 ± 0.02 ^ab^	0.03 ± 0.003 ^ab^

Results are presented as the mean (M) ± standard error of the mean (SEM). Values with different letters in the same column are significantly different according to the Tukey HSD test at *p* < 0.05.

**Table 3:** Catalase (CAT) activity in the larvae, pupae and midguts of honey bees exposed to RF-EMFs at a frequency of 900 MHz and electric field level of 70 mV m^−1^ (medium intensity—MI), 30 mV m^−1^ (low intensity—LI) and 1000 mV m^−1^ (high intensity—HI) at 2 weeks, 5 months and 1 year.

Sampling Time	Location	Electric Field Level (mV m^−1^)	CAT (Unit mg^−1^ _proteins_)		
			Larvae (N = 30)	Pupae (N = 20)	Midguts (N = 50)
2 weeks	MI	70	26.06 ± 2.54 ^abc^	22.76 ± 2.22 ^b^	85.43 ± 6.49 ^c^
	LI	30	33.46 ± 1.30 ^ab^	24.19 ± 1.89 ^b^	113.50 ± 5.69 ^abc^
	HI	1000	26.35 ± 1.40 ^abc^	22.97 ± 1.33 ^b^	119.62 ± 12.02 ^ab^
5 months	MI	70	20.98 ± 1.17 ^c^	38.23 ± 3.72 ^a^	109.65 ± 3.67 ^abc^
	LI	30	22.57 ± 2.63 ^bc^	39.59 ± 4.1 ^a^	122.68 ± 4.70 ^ab^
	HI	1000	36.10 ± 3.78 ^a^	28.22 ± 3.09 ^ab^	89.83 ± 6.29 ^c^
1 year	MI	70	24.18 ± 1.63 ^bc^	24.26 ± 1.53 ^b^	90.54 ± 2.00 ^bc^
	LI	30	36.07 ± 3.17 ^a^	28.52 ± 1.29 ^ab^	101.39 ± 5.09 ^abc^
	HI	1000	34.16 ± 3.39 ^ab^	24.13 ± 0.82 ^b^	130.87 ± 10.18 ^a^

Results are presented as the mean (M) ± standard error of the mean (SEM). Values with different letters in the same column are significantly different according to the Tukey HSD test at *p* < 0.05.

**Table 4:** Superoxide dismutase (SOD) activity in the larvae, pupae and midguts of honey bees exposed to RF-EMFs at a frequency of 900 MHz and electric field levels of 70 mV m^−1^ (medium electric field—MEF), 30 mV m^−1^ (low intensity—LI) and 1000 mV m^−1^ (high intensity—HI) at 2 weeks, 5 months and 1 year.

Sampling Time	Location	Electric Field Level (mV m^−1^)	SOD (Unit mg^−1^ _proteins_)		
			Larvae (N = 30)	Pupae (N = 20)	Midguts (N = 50)
2 weeks	MI	70	3.97 ± 0.51	3.70 ± 0.63 ^ab^	3.07 ± 0.30 ^bc^
	LI	30	4.45 ± 0.49	5.87 ± 1.23 ^ab^	4.13 ± 0.50 ^bc^
	HI	1000	5.29 ± 0.32	4.99 ± 1.03 ^ab^	3.63 ± 0.38 ^bc^
5 months	MI	70	3.61 ± 0.58	2.73 ± 0.63 ^ab^	4.69 ± 0.50 ^bc^
	LI	30	5.46 ± 0.37	2.11 ± 0.25 ^b^	3.71 ± 0.30 ^bc^
	HI	1000	4.64 ± 0.31	3.38 ± 0.50 ^ab^	6.52 ± 0.58 ^ab^
1 year	MI	70	4.14 ± 0.41	4.85 ± 0.77 ^ab^	2.56 ± 0.25 ^c^
	LI	30	5.41 ± 0.58	4.13 ± 0.66 ^ab^	6.08 ± 1.63 ^abc^
	HI	1000	3.75 ± 0.39	7.54 ± 1.75 ^ab^	9.34 ± 1.31 ^a^

Results are presented as the mean (M) ± standard error of the mean (SEM). Values with different letters in the same column are significantly different according to the Tukey HSD test at *p* < 0.05.

**Table 5:** Thiobarbituric acid reactive substance (TBARS) concentration in the larvae, pupae and midguts of honey bees exposed to RF-EMFs at a frequency of 900 MHz and electric field levels of 70 mV m^−1^ (medium intensity—MI), 30 mV m^−1^ (low intensity—LI) and 1000 mV m^−1^ (high intensity—HI) at 2 weeks, 5 months and 1 year.

Sampling Time	Location	Electric Field Level (mV m^−1^)	TBARS (Unit mg^−1^ _proteins_)		
			Larvae (N = 30)	Pupae (N = 20)	Midguts (N = 50)
2 weeks	MI	70	0.41 ± 0.07 ^c^	0.28 ± 0.02	1.78 ± 0.16 ^b^
	LI	30	0.40 ± 0.07 ^c^	0.27 ± 0.02	2.28 ± 0.10 ^ab^
	HI	1000	0.72 ± 0.16 ^ab^	0.18 ± 0.04	3.15 ± 0.40 ^a^
5 months	MI	70	0.69 ± 0.11 ^abc^	0.29 ± 0.02	1.70 ± 0.16 ^b^
	LI	30	0.88 ± 0.14 ^abc^	0.22 ± 0.07	1.72 ± 0.15 ^b^
	HI	1000	1.02 ± 0.15 ^ab^	0.19 ± 0.04	2.44 ± 0.24 ^ab^
1 year	MI	70	0.47 ± 0.08 ^bc^	0.21 ± 0.03	2.50 ± 0.30 ^ab^
	LI	30	1.09 ± 0.16 ^a^	0.21 ± 0.05	2.63 ± 0.08 ^ab^
	HI	1000	1.26 ± 0.12 ^a^	0.15 ± 0.02	3.00 ± 0.15 ^a^

Results are presented as the mean (M) ± standard error of the mean (SEM). Values with different letters in the same column are significantly different according to the Tukey HSD test at *p* < 0.05.

## Data Availability

The data presented in this study are available on reasonable request from the corresponding author.
